# The microRNA-451a/chromosome segregation 1-like axis suppresses cell proliferation, migration, and invasion and induces apoptosis in nasopharyngeal carcinoma

**DOI:** 10.1080/21655979.2021.1975018

**Published:** 2021-09-13

**Authors:** Yi Luo, Xiu Qu, Dan Kan, Binlin Cai

**Affiliations:** a Department of Otorhinolaryngology, Affiliated Puren Hospital of Wuhan University of Science and Technology, Wuhan, China; b Department of Pain Treatment, Affiliated Puren Hospital of Wuhan University of Science and Technology, Wuhan, China

**Keywords:** miR-451a, CSE1L, cell proliferation, cell viability, cell invasion, nasopharyngeal carcinoma

## Abstract

MicroRNA-451a (miR-451a) has been implicated in the initiation and progression of multiple cancers. However, the regulatory mechanisms underlying its function in nasopharyngeal carcinoma (NPC) are poorly understood. Thus, we investigated in detail the role of the microRNA-451a/chromosome segregation 1-like (miR-45a/CSE1L) axis and its regulatory mechanism in NPC. We examined the levels of miR-451a and CSE1L in NPC, and assessed the effects of miR-451a and CSE1L on NPC by cell functional experiments. Furthermore, we elucidated the direct regulatory effect of miR-451a on *CSE1L* by the luciferase reporter assay, RNA pull-down assay, and RNA immunoprecipitation and validated our observations by calculating the Pearson’s correlation coefficient. We found that miR-451a was down-regulated in NPC cells, and its over-expression attenuated cell proliferation, migration, and invasion, and tumor growth in 5–8 F and SUNE-1 cells and promoted apoptosis. Moreover, *CSE1L* was the direct gene target of miR-451a, and its over-expression abrogated miR-451a-dependent inhibition of malignancy in 5–8 F and SUNE-1 cells. The Pearson’s correlation coefficient indicated a negative correlation between *CSE1L* and miR-451a. miR-451a serves as a tumor suppressor and targets *CSE1L*. miR-451a suppresses *CSE1L* expression, thereby reducing proliferation, invasion, and migration and increasing apoptosis of NPC cells.

## Background

Nasopharyngeal carcinoma (NPC) originates from nasopharyngeal epithelial cells and is characterized by racial and geographical disparities [1]. This unbalanced distribution mainly results from genetic susceptibility, viral infection, and lifestyle risks [2–4]. An estimated 129,000 new NPC cases were reported worldwide in 2019 [5]. Despite great improvements in treatment approaches, patients are frequently diagnosed with NPC at an advanced stage, when the cancer is highly aggressive, thereby reducing their chances of survival. Thus, better insights into the genetic heterogeneity and tumor biology of NPC might facilitate timely diagnosis and therapy in the future.

MicroRNAs (miRNAs) are approximately 20-nucleotide long single-stranded RNA molecules [6]. Although they do not encode proteins, they can regulate gene expression by degrading or suppressing the translation of target mRNAs. Thus, miRNAs can alter several cellular functions, leading to the development of various diseases. Moreover, compelling evidences have indicated that miRNAs have potential functions in pan-cancers, including NPC [7,8]. For example, over-expression of miR-330-3p in ovarian cancer cells inhibits cell proliferation, migration, and invasion and halts ovarian cancer progression [9]. On the other hand, miR-1269b in gastric cancer is linked to tumor enlargement and lymph node metastasis, and inhibits malignancy in gastric cancer cells [10]. The tumor suppressor miR-3188 suppresses cell proliferation and sensitizes NPC cells to chemotherapeutics [11]. Reportedly, miR-451a has tumor suppressor activities in various cancers; for instance, it sensitizes lung tumors to chemotherapy and inhibits epithelial-to-mesenchymal cell transition in lung cancer [12]. Moreover, miR-451a inhibits cell proliferation and migration and in vitro cell invasion in papillary thyroid carcinoma [13]. Remarkably, miR-451a suppresses tumorigenesis by regulating cell apoptosis and angiogenesis in hepatocellular carcinoma [14]. However, the biological significance of miR-451a in NPC progression has not been investigated yet.

Chromosome segregation 1-like (*CSE1L*) encodes a cellular apoptosis susceptibility protein and orchestrates multiple biological processes, such as apoptosis, cell proliferation, and chromosome alignment and congression [15]. As *CSE1L* regulates mitosis and cell survival, it is considered to be a putative oncogene and is highly amplified in pan-cancers [16–18]. Furthermore, previous studies have revealed that high expression of *CSE1L* positively correlates with tumor invasion and distant metastasis in different cancers [18–20]. Additionally, amplification of the *CSE1L* oncogene has been detected in NPC tissues and is associated with poor patient prognosis [21,22]. However, its function in NPC remains unclear.

In the current study, we aim to understand the role of miR-451a in propagating malignancy in NPC. Therefore, we have used bioinformatics analysis to reveal key roles of miR-451a and *CSE1L* in NPC. In addition, we have examined the potential regulatory functions of miRNA-451a via *CSE1L* in malignant NPC cells. Our findings highlight the potential of miR-451a/*CSE1L* as a novel target for treating patients with NPC.

## Methods

### Bioinformatics analysis

We retrieved the GSE118613 dataset from Gene Expression Omnibus (GEO), containing miRNA profiles obtained from NPC tumor and non-tumor samples. Subsequently, we screened these profiles to identify the miRNAs that were down-regulated in NPC with parameters kept as P < 0.05 and logFC≤-1.5. Additionally, we retrieved the GSE12452 dataset from GEO, consisting of mRNA profiles obtained from NPC tumor and non-tumor samples. We also screened these profiles to identify mRNAs that were up-regulated in NPC and kept the parameters as P < 0.01 and logFC ≥ 1.5. Target genes of miR-451a were predicted using the starBase algorithm (http://starbase.sysu.edu.cn/).

### Human tissue samples and cell culture conditions

We acquired 32 pairs of tumor tissues and normal adjacent tissues from 32 NPC patients at our hospital. All patients were diagnosed with NPC by magnetic resonance imaging and endoscopy. Signed informed consent was obtained from all patients, and our study was approved by the ethics committee of our hospital. Clinical characteristics of these patients are listed in Table 1.

**Table 1.** Association of miR-451a and clinicopathological features in patients with NPC

Characteristics	N = 40	miR-451a expression		P
		Low (n = 16)	High (n = 16)	
Age (years)				0.285
≤45	14	5	9	
>45	18	11	7	
Gender				0.394
Male	22	11	14	
Female	10	5	2	
TNM stage				0.001
I+ II	12	1	11	
III+IV	20	15	5	
Local or distant metastasis				0.023
No	21	7	14	
Yes	11	9	2	
Smoking history				0.473
No	19	8	11	
Yes	13	8	5	
Differentiation				0.113
Undifferentiated	9	7	2	
Low and moderately differentiated	23	9	14	

We preserved the tumor tissues at −80°C before performing RNA extraction. Human NPC cell lines, 6–10B, 5–8 F, and SUNE-1, and the human immortalized nasopharyngeal epithelial cell line NP69 were obtained from Shanghai Kenqiang Company (China). All cells were maintained in Dulbecco’s modified Eagle’s medium containing 10% fetal bovine serum and 1% penicillin/streptomycin at 37°C in a 5% CO_2_ incubator.

### Cell transfection

Synthetic miR-451a mimics and scrambled RNA (negative control, NC) were obtained from Suzhou GenePharma. Both synthetic miR-451a mimics and NC were transfected into 1 × 10^3^ 5–8 F and SUNE-1 cells using Lipofectamine 2000 (Thermo Fisher Scientific, China) as per the manufacturer’s instructions. To produce cells that over-expressed *CSE1L*, we transfected 5–8 F and SUNE-1 cells with the overexpression vector *pcDNA 3.1*-CSE1L (OE) and the empty vector (OE-NC), purchased from Sangon Biotech, using Lipofectamine 3000 (Thermo Fisher Scientific, USA). We calculated the transfection efficiency by quantitative reverse transcription polymerase chain reaction (qRT-PCR) after 48 h of transfection.

### qRT-PCR

We extracted RNA from tissues and cells using the TRIzol reagent (Invitrogen). Subsequently we performed cDNA synthesis using the ImProm-II reverse transcription kit (Promega, USA) by mixing 1 μg of RNA (from tumor and transfected cells each) to 0.5 µL oligodT primers and 0.5 µL miRNA-specific stem-loop primers, respectively. SYBR Green Supermix (Bio-Rad, USA) was used for quantifying the mRNA expression of miR-451a and *CSE1L*. We used U6 and *GAPDH* as internal references and determined the mRNA expression levels of miR-451a and *CSE1L* using the 2^−ΔΔCT^ method [23]. The sequences for PCR primers used in this study are shown in Table 2.

**Table 2.** The sequence of PCR primers used in this study

Gene name	Primer type	Sequence
miR-451a	Forward	5ʹ-TGGCCGTTACCATTACTGAGTT-3’
	Reverse	5ʹ-GCGACGAGCAAAAAGCTTGT-3’
CSE1L	Forward	5ʹ‐TTTTGAGTTACCCGAAGA‐3ʹ
	Reverse	5ʹ‐TTGTGAAGTGACTGTGCC‐3ʹ
GAPDH	Forward	5ʹ‐GTCAGCCGCATCTTCTTTTG‐3ʹ
	Reverse	5ʹ‐GCGCCCAATACGACCAAATC‐3ʹ
U6	Forward	5ʹ-CTCGCTTCGGCAGCACA-3′
	Reverse	5ʹ-AACGCTTCACGAATTTGCGT-3’

### Western blotting

Protein levels were measured as previously described [24]. At 48 h post-transfection, 5–8 F and SUNE-1 cells were lysed using the radioimmunoprecipitation assay buffer (lysis buffer; Abcam, China). We quantified the supernatants using a bicinchoninic acid protein concentration kit (Beyotime, China). Subsequently, proteins (20 µg) were separated with 12% sodium dodecyl sulfate polyacrylamide gel electrophoresis and were electro-transferred onto a polyvinylidene fluoride membrane. The membrane was incubated with 5% skimmed milk for 1 h at room temperature before being incubated with rat anti-CSE1L (1:1000, Cat. no. ABIN560494, Beijing 4A Biotech Co, Ltd., China), and rat anti-GAPDH (1:1000, Cat. no. AF1186, Beyotime, China) at 4°C for 24 h. The following day, the blot was probed with a mouse anti-rabbit horseradish peroxidase–conjugated secondary antibody (1:1000, Cat. no. 4060–01, Southern Biotech, USA), and protein bands were visualized by chemiluminescence using ECL (Beyotime, China).

### Cell counting kit-8 (CCK-8) assay

We assessed the viability of NPC cells at 48 h post-transfection using the CCK-8 kit (4A Biotech Co. Ltd. China), as previously described [10]. Briefly, 2 × 10^3^ cells/well were seeded in a 96-well plate. Cells were cultured for 24 h, 48 h, 72 h and 96 h, following which 20 μL of CCK-8 was added to each well. Cells were incubated for additional 2 h, and the optical density (OD) of each well was measured at 450 nm using a spectrophotometer (BioTek, USA).

### Bromodeoxyuridine (BrdU) cell proliferation assay

This assay was performed as described previously [25]. We measured the cell proliferation rate at 48 h post-transfection in the 5–8 F and SUNE-1 cell lines using the BrdU cell proliferation assay kit (Frdbio, China) as per the manufacturer’s instructions. In brief, 1 × 10^3^ cells/well were seeded in 24-well plates to achieve 60–70% confluency. Thereafter, cells were cultured in 10 μl BrdU solution for 4 h and were fixed with 5% paraformaldehyde for 20 min. Subsequently, cells were permeabilized in 0.5% Triton X-100 and incubated with a horseradish peroxidase-conjugated mouse anti-BrdU monoclonal antibody (1:1000) overnight at room temperature. Following this, 100 μL of a horseradish peroxidase substrate was added to the cells, and the cell plates were kept in the dark for 30 min. The OD was measured at 450 nm using a microplate reader (Biobase, China).

### Flow cytometry analysis

At 48 h post-transfection, 5–8 F and SUNE-1 cells were collected, and cellular apoptosis was detected by the Annexin V-FITC/PI Apoptosis Detection kit (BD Biosciences, USA), performed as per the manufacturer’s protocol. Briefly, cells were diluted with 400 μL of Annexin V binding buffer. Subsequently, they were incubated with 5 μL of FITC and 5 μL of propidium iodide and kept in the dark for 15 min at room temperature. Cells were analyzed using a FACSAria flow cytometer (BD Biosciences) [26].

### Wound healing assay

A wound healing assay was performed as described previously [27]. At 48 h post-transfection, 5 × 10^3^ cells/well were seeded in 6-cm dishes to achieve 80–90% confluency. A pipette tip (10 μL) was used to produce a 400 μm gap in the confluent monolayer. After removing the detached cells, the adherent cells were cultured for an additional 24 h, following which they were fixed with 3.7% paraformaldehyde and stained with 1% crystal violet. Cell migration was observed under a phase-contrast microscope and the cell migration rate was calculated by Image J; it was calculated as (0 h width of gap-24 h gap width)/0 h gap width×100%.

### Transwell invasion assay

Cell invasiveness was assessed using a Transwell insert. A Matrigel-precoated transwell insert was plated with 5 × 10^3^ cells at 48 h post-transfection. This insert was added on a lower insert, containing 600 mL fresh medium. Cells were cultured for 24 h, following which the upper chamber was removed and the noninvasive cells were wiped out with a cotton swab. Next, the cells were fixed with formaldehyde for 20 min and subsequently stained with 0.1% crystal violet for 10 min. Invasive cells were visualized by phase-contrast microscopy, and the rate of invasiveness was calculated as described previously [28].

### Tumor xenograft model

All our experiments on animals were approved by the Institutional Animal Care and Use Ethics Committee of our hospital. Ten male BALB/c nude mice aged 4–6 weeks were purchased from the Medical Experimental Animal Center of Guangdong Province (Guangzhou, China). We suspended 1 × 10^6^ of 5–8 F cells that stably over-expressed miR-451a or the NC in 200 μL of PBS. Subsequently, these cells were injected subcutaneously into the dorsal side of the nude mice. We measured the tumor size and calculated the tumor volume every 6 days. After 30 days, the mice were euthanized, and the tumors were dissected and weighed [29].

### Dual-luciferase reporter assay

To further verify whether the 3′ untranslated region (3΄ UTR) of *CSE1L* mRNA contained the miR-451a binding site, we inserted fragments of the wild-type 3′ UTR and mutant 3′ UTR regions into the pmirGLO luciferase reporter plasmid (Promega, USA). Subsequently, we cloned these fragments and obtained pmirGLO-CSE1L3′-UTR-WT (WT) and pmirGLO-CSE1L3′-UTR-MUT (MUT). Next, 1 × 10^5^ cells were co-transfected with the WT or MUT vectors and with the synthetic miR-451a mimic or NC. At 48 h post-transfection, cells were lysed, and luminescent signals were detected using a Dual Luciferase Reporter Assay kit (Promega, USA). Relative luciferase activity was presented as firefly luciferase/Renilla luciferase [30].

### RNA pull-down assay

The assay was performed as described previously [31]. Biotin-labeled miR-451a (Bio-miR-451a) and negative control (Bio-NC) were synthesized by GenePharma (Shanghai, China). Cells were transfected with Bio-NC and Bio-miR-451a probes for 48 h, following which they were lysed in lysis buffer and incubated with Dynabeads M-280 streptavidin (Invitrogen). Each streptavidin-bound biotin-labeled RNA was eluted using magnetic beads; the eluted RNA was analyzed by qRT-PCR.

### RNA immunoprecipitation (RIP)

We performed RIP for the 5–8 F and SUNE-1 cell lines using the Magna RIP RNA-Binding Protein Immunoprecipitation Kit (Millipore, USA). Briefly, cells were lysed with the RIP buffer, and after centrifugation the lysate was collected and re-suspended. Subsequently, it was incubated with Ago2 or IgG antibody-conjugated magnetic beads for 30 min. After isolating the precipitated RNA, the enriched CSE1L mRNA and miR-451a were detected by qRT-PCR [32].

### Statistical analysis

We statistically analyzed our data using GraphPad Prism version 9.0; these results were presented as mean±standard deviation (SD). All experiments were performed in triplicates. Either One-way ANOVA or Two-way ANOVA was used to analyze data between multiple groups, whereas the Paired student’s t-test was performed to analyze data between two groups. The Pearson’s correlation coefficient was calculated to correlate the expression of miR-451a with that of *CSE1L*. Statistical relevance was defined as p < 0.05.

## Results

In this study, we investigated the effects of miR-451a and *CSE1L* on cell viability, proliferation, migration, invasion, and tumor growth in NPC. We observed that miR-451a expression was down-regulated, whereas that of *CSE1L* was up-regulated in NPC. Moreover, we observed that over-expression of miR-451a suppressed malignancy in NPC cells. Since *CSE1L* is a direct target of miR-451a, its overexpression could eliminate miR-451a-dependent malignancy in 5–8 F and SUNE-1 cells.

### *Identification of miR-451a and* CSE1L *as the genes of interest in NPC*

**Figure 1.**
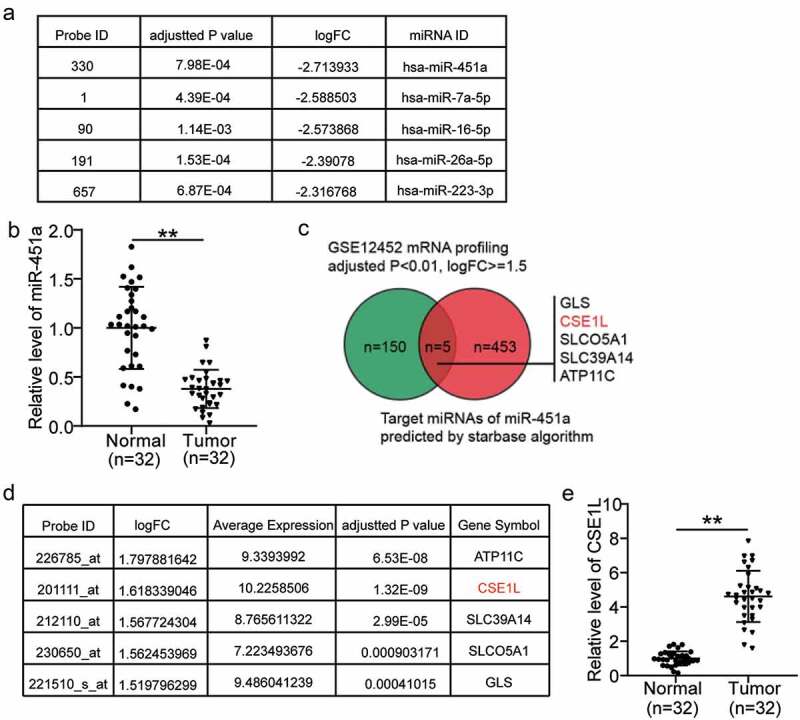
The selection of miR-451a and CSE1L as our study objects in NPC. (a). The top 5 most downregulated miRNAs in NPC. Data obtained from GSE118613 analysis. Criteria: adjusted P < 0.05, logFC≤-1.5. (b). miR-451a expression in NPC tissues and non-cancerous tissues by RT-qPCR. (c). Venn diagram showing the intersection between the predicted target genes of miR-451a by starbase and the significantly upregulated genes from GSE12452 data analysis (adjusted P < 0.01, logFC≥1.5). (d). The expression of the five genes in GSE12452 data analysis. (e). CSE1L expression in NPC tissues and non-cancerous tissues by RT-qPCR

Upon analyzing the miRNA expression profiles in the GSE118613 dataset, we found that miR-451a was the most significantly down-regulated miRNA in NPC (Figure 1a). It has been reported to be a tumor suppressor in other human cancers, but not in NPC [13,33–38]. Remarkably, we found that miR-451a expression was significantly down-regulated in the NPC tumor tissues compared to that in the normal adjacent tissues (Figure 1b). Subsequently, we identified 458 unique downstream effectors of miR-451a using starbase algorithm. We also analyzed the mRNA expression profiles of NPC, retrieved from the GSE12452 dataset. We identified 155 mRNAs that were significantly up-regulated in NPC, among which 5 were also predicted to be the downstream effectors of miR-451a: *GLS, CSE1L, SLCO5A1, SLC39A14, and ATP11C* (Figure 1c). From the GSE12452 dataset, we also found that *CSE1L* had the highest up-regulated mRNA expression in NPC (average expression level = 10.22; Figure 1d). In addition, our data suggested that the expression levels of ATP11 C, CSE1L, SLC39A14, SLCO5A1, and GLS were higher in tumor tissues than in normal tissue by a factor of 3.4, 4.7, 2.8, 1.7 and 3.6, respectively. The most significant up-regulation was observed in that of *CSE1L* expression; therefore, we focused on it in the subsequent experiments (Supplementary Figure 1a–d, and Figure 1e). Studies have reported CSE1L to act as a tumor suppressor in colorectal cancer by functioning in ceRNA networks [39,40]. Nonetheless, the roles of miR-451a and CSE1L have not yet been investigated in NPC, and their interactions have not yet been studied in any human cancers.

### miR-451a expression attenuates cellular proliferation and promotes apoptosis in NPC

**Figure 2.**
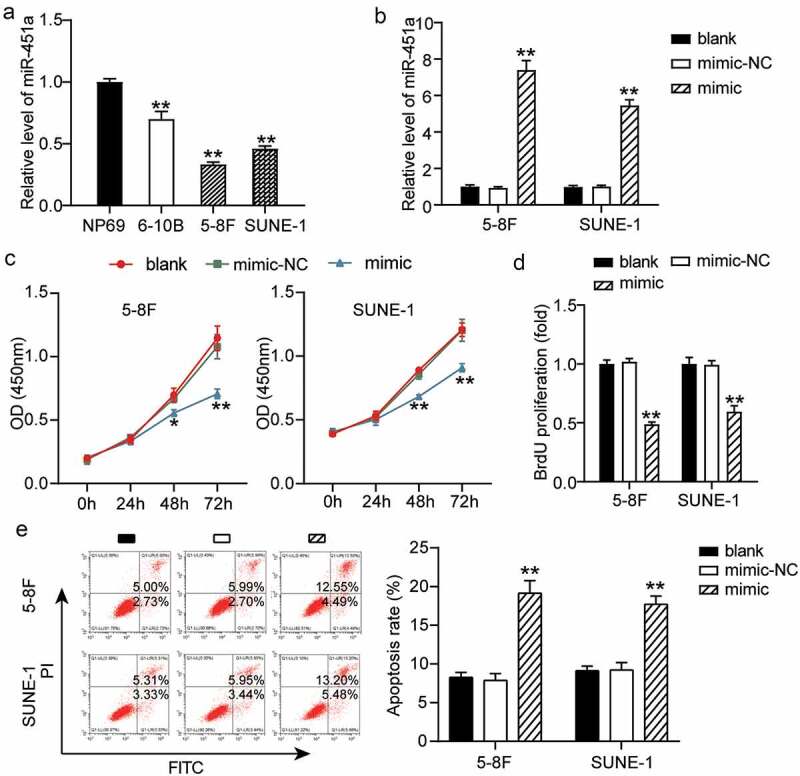
MiR-451a could inhibit the NPC cells proliferation. (a). mRNA levels of miR-451a in NP69, 6–10B, 5–8 F and SUNE-1 cells. **P < 0.001 vs.NP69. (b). The efficiency of transfected miR-451a mimic or negative control (NC) at 48 h was validated by RT-qPCR. (c). CCK8 assay revealing the suppression of miR-451a overexpression on the viability of 5–8 F and SUNE-1 cells. (d). BrdU assay demonstrated the inhibiting effect of miR-451a on the proliferation of 5–8 F and SUNE-1 cells. E. Flow cytometry assay showing the promoting effect of miR-451a on the apoptosis rate of 5–8 F and SUNE-1 cells. *P < 0.05, **P < 0.001 vs. blank

Previous observations have revealed that differential expression of miR-451a is associated with diverse types of cancers [33,37]. We investigated the clinical significance of miR-451a expression in NPC. To this end, we grouped NPC patients into miR-451a low expression (n = 16) and high expression (n = 16) groups based on the median value of the miR-451a expression levels. Subsequently, the correlation between the clinicopathological features of NPC patients and miR-451a expression levels was evaluated. As shown in Table 1, a low miR-451a expression was significantly associated with an advanced tumor, nodes, and metastasis (TNM) stage and with local or distant metastasis in NPC. The miR-451a expression profiles of the cell lines 6–10B, 5–8 F, and SUNE-1 revealed lower miR-451a expression in these cell lines than that in the NP69 cell line (Figure 2a). Hence, we chose 5–8 F and SUNE-1 cell lines, containing the lowest miR-451a expression levels, to examine whether synthetic miRNA analogs altered cellular processes in NPC. We transfected 5–8 F and SUNE-1 cells with synthetic miRNA mimics and NC and generated a cell transfection curve. Our results showed that in both the cell lines, expression levels of the mimic increased with prolonged transfection and stabilized at 48 h (Supplementary Figure S1); at this time poin,t a five-fold or greater increase in the miR-451a expression levels was observed (Figure 2b). Furthermore, we observed that upon over-expression of the miR-451a mimic in 5–8 F and SUNE-1 cells, cell viability was significantly impaired in both the cell lines, as assessed by the CCK-8 assay (Figure 2c). Moreover, we also observed that upon miR-451a over-expression, cell proliferation was impaired in these cell lines, as evaluated by the BrdU assay (Figure 2d). Additionally, we observed that NPC cells transfected with the synthetic miR-451a mimic (Figure 2e) had a higher apoptotic rate than the ones transfected with the NC. These observations suggested that miR-451a over-expression suppressed cell proliferation and promoted apoptosis in the 5–8 F and SUNE-1 cell lines.

### Over-expression of miR-451a suppresses cell migration and invasion in vitro and suppressed tumor growth in vivo in NPC

**Figure 3.**
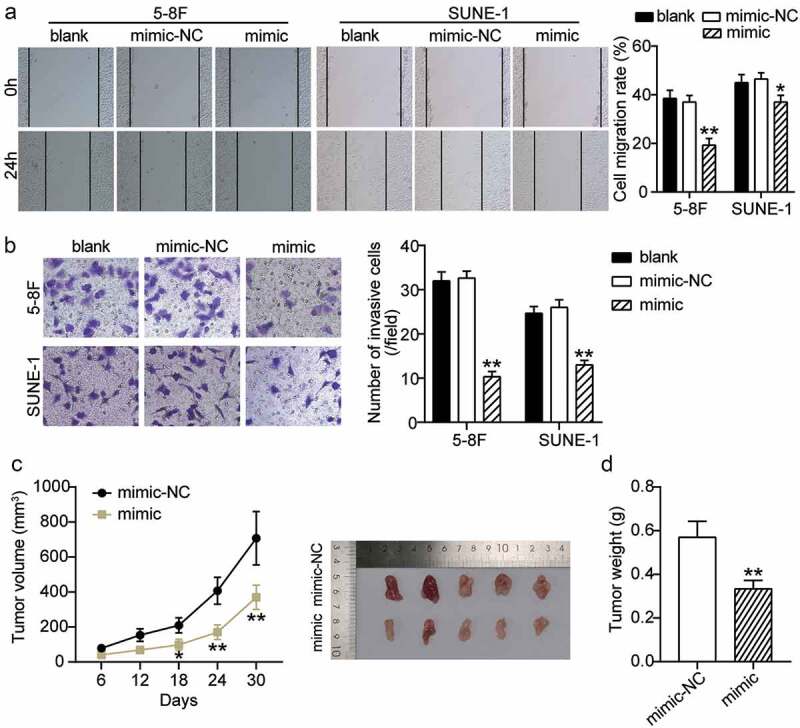
MiR-451a negatively regulates the migration, invasion and xenograft tumor growth of NPC cells. 5–8 F and SUNE-1 cells were transfected with miR-451a synthetic miRNA analogs. The migratory and invasive capacities of both cells were evaluated by the wound-healing assay (a) and transwell assay (b). (c) Representative images of tumors formed and the growth curves of tumor volume. (d) Tumor weight. *P < 0.05, **P < 0.001 vs. blank or mimic-NC

We assessed the effect of miR-451a over-expression on cell migration and invasion in the 5–8 F cand SUNE-1 cell lines. The wound healing assay indicated a significantly reduced cell migration rate in 5–8 F and SUNE-1 cells transfected with the synthetic miR-451a mimic compared to cells transfected with the blank control and NC (Figure 3a). Additionally, our transwell assay results indicated that cell invasiveness was reduced in cells over-expressing miR-451a (Figure 3b). Since miR-451a inhibited the survival of NPC cells in vitro, we examined whether over-expression of miR-451a could affect tumorigenesis in vivo. As shown in Figure 3c, over-expression of miR-451a in the tumor xenograft model significantly reduced the tumor growth and volume. In addition, the tumor weight in mice that over-expressed the synthetic miR-451a mimic was significantly lower than that in mice that expressed the NC (Figure 3d). These observations suggested that miR-451a inhibits malignant progression in NPC in vivo and in vitro.

### CSE1L acts as a direct target of miR-451a

**Figure 4.**
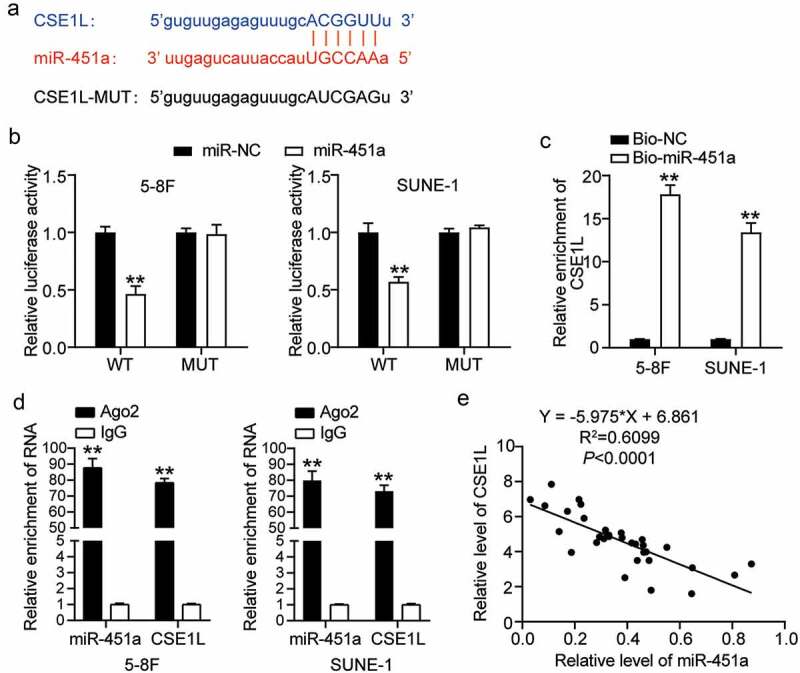
CSE1L is a direct target of miR-451a. (a). starBase analysis predicted that 3′UTR sequence of CSE1L contained the complementary sequence of miR-451a. (b). Luciferase activity of CSE1L with wild type (WT) or mutant (MUT) 3ʹUTR in 5–8 F and SUNE-1 cells. **P < 0.001 vs. miR-NC. (c). RNA pull-down assay was performed to analysis the interaction between CSE1L and miR-451a in 5–8 F and SUNE-1 cells transfected with miR-451a mimic and miRNA-NC. **P < 0.001 vs. Bio-NC. (d). The expression of miR-451a and CSE1L extracted by RIP assay was evaluated in samples bound to Ago2 or IgG. **P < 0.001 vs. IgG. E. Pearson assay showed a strong negative correlation (R^2^ = 0.7123) between miR-451a level and CSE1L expression in NPC tissues

miRNAs are known to execute their functions via the miRNA-target gene regulatory network [6]. We identified *CSE1L* among the putative target genes of miR-415a, which was reported to be associated with NPC tumorigenesis [41]. Moreover, we demonstrated that the direct binding site of miR-451a was located on the 3΄ UTR region of the CSE1L mRNA (Figure 4a). For instance, we observed significantly impaired CSE1L-WT-mediated luciferase activity in cells that had been transfected with the synthetic miR-451a mimic; however, no such change was observed in CSE1L-MUT-mediated luciferase activity (Figure 4b). To validate the interaction between miR-451a and *CSE1L*, we measured the enrichment levels of CSE1L mRNA and biotin-labeled miR-451a by the RNA pull-down assay. As depicted in Figure 4c, *CSE1L* mRNA levels in both 5–8 F and SUNE-1 cells transfected with Bio-miR-451a were 12-fold higher than in the cells transfected with Bio-NC. Moreover, RIP results revealed that cells treated with anti-Ago2 molecules were more enriched in miR-451a and *CSE1L* than those treated with anti-IgG molecules (Figure 4d). In addition, Pearson’s correlation coefficient revealed a strong negative correlation between *CSE1L* expression and miR-451a in NPC tissues (Figure 4e).

### *Overexpression of* CSE1L *alleviates miR-451a-mediated inhibition of proliferation and promotion of apoptosis.*

**Figure 5.**
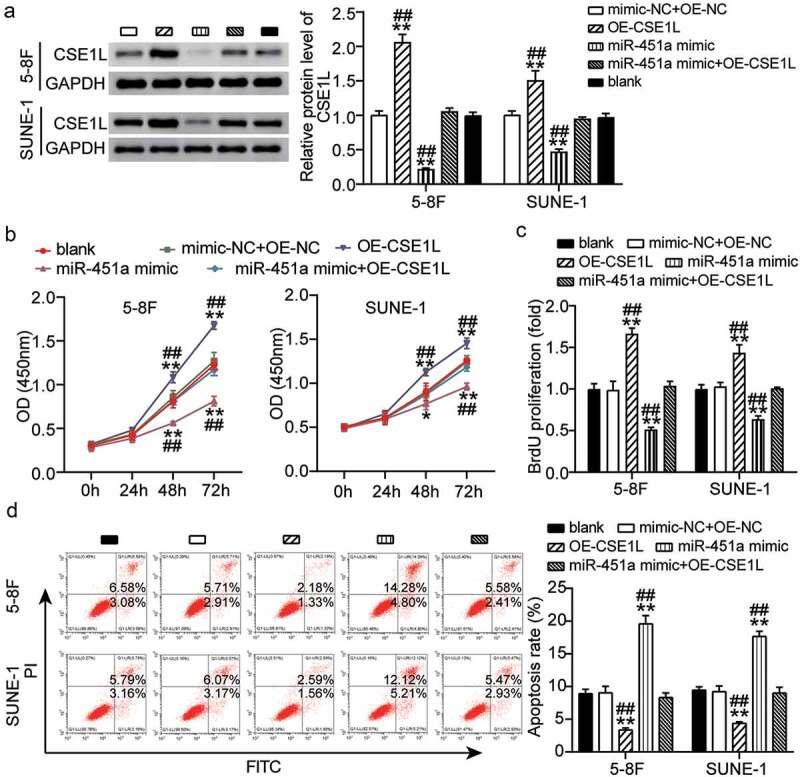
Ecotopic expression of CSE1L offsets the modulation of miR-451a on proliferation of NPC cells. (a). The expression of CSE1L in 5–8 F and SUNE-1 cells was detected by western blot. (b). CCK8 assay examining the viability of 5–8 F and SUNE-1 cells following transfection with *pcDNA 3.1*-CSE1L (OE), miR-451a mimic, OE-NC + mimic-NC, OE + mimic. (c). BrdU assay was performed to quantify the proliferation of 5–8 F and SUNE-1 cells following transfection with OE, miR-451a mimic, OE-NC + mimic-NC, OE, OE + mimic. (d). Flow cytometry assay was performed to quantify the apoptosis rate of 5–8 F and SUNE-1 cells following transfection with OE, miR-451a mimic, OE-NC + mimic-NC, OE + mimic. **P < 0.001 vs. blank; ^##^P < 0.001 vs. OE + mimic

*CSE1L* is recognized as an oncogene that is commonly amplified in NPC [21] and is associated with proliferation, apoptosis, invasion, and metastasis [22]. We observed that expression of *CSE1L* was rescued in NPC cells that were co-transfected with the synthetic miR-451a mimic and *pcDNA 3.1*-CSE1L, as evident by our western blot results (Figure 5a). Additionally, CCK-8 analysis indicated that 5–8 F and SUNE-1 cells transfected with OE-CSE1L had increased cell viability, partially eliminating the inhibitory effect that miR-451a overexpression had on cell viability (Figure 5b). Consistently, BrdU assay results indicated that ectopic expression of *CSE1L* significantly promoted cell proliferation in both 5–8 F and SUNE-1 cells, reversing the inhibitory effect of miR-451a overexpression on cell proliferation (Figure 5c). In addition, *CSE1L* up-regulation inhibited apoptosis in NPC cells, and to a certain extent, weakened the pro-apoptotic effect of the synthetic miR-451a mimic (Figure 5d). These observations implied that *CSE1L* over-expression could rescue the inhibition of cell proliferation mediated by miR-451a overexpression.

### *Ectopic expression of* CSE1L *alleviates miR-451a mimic-mediated inhibition of cell migration and invasion in NPC*

**Figure 6.**
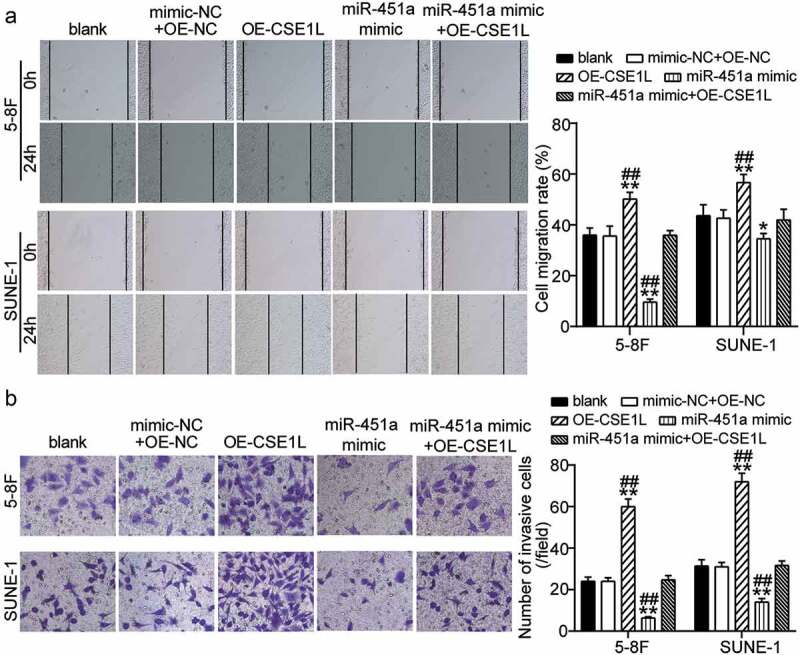
CSE1L overexpression restores the migration and invasion impairment of NPC cells induced miR-451a mimic. (a). In vitro wound healing analysis of NPC cells migration after transfected with *pcDNA 3.1*-CSE1L (OE), miR-451a mimic, OE-NC + mimic-NC, OE + mimic at 0 and 48 h. (b). In vitro transwell assay was performed to examine the invasion of NPC cells following transfection with OE, miR-451a mimic, OE-NC + mimic-NC, OE + mimic. **P < 0.001 vs. blank; ^##^P < 0.001 vs. OE + mimic

Subsequently, we analyzed whether *CSE1L* overexpression affected cell migration and invasion in miR-451a-over-expressing 5–8 F and SUNE-1 cells. Remarkably, the area of the healed wound increased upon *CSE1L* over-expression in both cell lines and reduced post transfection with the synthetic miR-451a mimic. This indicated that wound healing was effectively rescued in cells over-expressing *CSE1L* (Figure 6a). Similarly, we also observed an increase in cell invasiveness upon *CSE1L* over-expression. Moreover, inhibition of cell invasion was rescued in cells co-transfected with miR-451a mimic and *pcDNA 3.1*-CSE1L in both the cell lines (Figure 6b). These findings suggested that *CSE1L* over-expression offsets the miR-451a-mediated suppression of cell migration and invasion in NPC in vitro.

## Discussion

A new understanding of NPC etiopathological mechanisms offers novel insights into carcinogenesis. Thus, elucidating the function of miRNAs in cell proliferation, migration, and invasion can broaden our knowledge on NPC progression. In this study, we detected that miR-451a expression levels were reduced in NPC cell lines. Subsequent in vitro assays indicated that over-expression of miR-451a impaired cell proliferation, migration, and invasion in NPC. This impairment was at least partially controlled by *CSE1L*. Over-expression of *CSE1L* abrogated miR-451a-mediated inhibition of malignancy in NPC. In addition, the negative connection between miR-451a and CSE1L mRNA levels in NPC further validated our findings that *CSE1L* is a direct target of miR-451a. Collectively, our study revealed for the first time that miR-451a can inhibit cell proliferation, migration, and invasion and can promote apoptosis by targeting *CSE1L*; thus, it has an anticancer role in NPC.

Currently, pleotropic evidence has demonstrated that miR-451a abrogates malignant characteristics, such as cell proliferation, apoptosis, and epithelial-to-mesenchymal transition, that drive cancer progression and unfavorable treatment responses in patients [12,35,42,43]. For example, miR-451a suppresses disease aggressiveness in papillary thyroid cancer [35]. Moreover, its down-regulated expression in multiple myeloma promoted cancer relapse in patients [44]. Furthermore, exogenous miR-451a expression can inhibit drug resistance and restore drug sensitivity in patients with acute myeloid leukemia [45]. However, thus far, the function of miR-451a in NPC remains unknown. The data presented here demonstrate that miR-451a is down-regulated in 5–8F and SUNE-1 cell lines compared to that in the NP69 cell line. Subsequent cell functional assays demonstrated that over-expression of miR-451a significantly impaired cell proliferation, migration, and invasion in both the cell lines. Therefore, our observations are congruent with those of previous studies that proposed that miR-451a is a tumor suppressor and that dysregulation of miR-451a may promote cancer progression.

Given the conserved regulatory functions of miRNA, miR-451a has been shown to post-transcriptionally modulate diverse genes implicated in tumor initiation and progression [33,34,37,46]. Therefore, we used microRNA target predictions starBase and identified gene targets associated with cell proliferation and apoptosis in NPC. One such predicted target, *CSE1L*, has been demonstrated to modulate tumor malignancy in various cellular models [15,18,20,47], and it is highly expressed in NPC cells [21,22,41]. However, it is not clear whether interaction between miR-451a and *CSE1L* accounts for the tumor-suppressive effects observed in NPC. Here, we identified *CSE1L* as a direct target of miR-451a using a luciferase reporter assay. In addition, we found that over-expression of miR-451a in both 5–8 F and SUNE-1 cell lines significantly reduced the CSE1L mRNA level. We also observed that *CSE1L* and miR-451a exhibited contrasting cellular functions in NPC. Furthermore, we validated that over-expression of *CSE1L* in both 5–8 F and SUE-1 cells increased cell proliferation, migration, and invasion, and decreased apoptosis. However, the oncogenic role of *CSE1L* was offset by exogenous expression of the synthetic miR-451a mimic. Therefore, our results reiterate that miR-451a has anti-cancer properties in NPC, concurring with those of previous studies.

However, there were multiple limitations to this study. To this end, we will be validating our observations in in vivo models and in clinical experiments. Subsequently, as we have focused on only direct targets of miR-451a in the current study, we aim to elucidate the entire regulatory network of miR-451a in NPC in our future studies.

## Conclusions

Collectively, our present data substantiate that miR-451a exerts tumor-suppressive functions by targeting *CSE1L* to hinder cell proliferation, migration, and invasion in NPC. Thus, we deduce that the miR-451a/*CSE1L* axis provides novel insights into targeted cancer therapeutics for NPC patients.

## Supplementary Material

Supplemental Material

## Data Availability

The datasets used and analyzed during the current study are available from the corresponding author on reasonable request.
