# Supplementary material for: The microRNA-451a/chromosome segregation 1-like axis suppresses cell proliferation, migration, and invasion and induces apoptosis in nasopharyngeal carcinoma
Source: Bioengineered. 2021 Sep 13;12(1):6967–80. doi: 10.1080/21655979.2021.1975018 (PMC8806603; doi:10.1080/21655979.2021.1975018)
Supplement: Supplemental Material [file KBIE_A_1975018_SM2662.zip › supplementary/Supplementary Figure legend.docx]

**Supplementary Figure 1. The expression of ATP11C, SLC39A14, SLCO5A1, GLS, and miR-451a.** A-D. The expression of ATP11C, SLC39A14, SLCO5A1, and GLS in NPC tissues and non-cancerous tissues by RT-qPCR. ^**^*P*<0.001. E. RT-qPCR was used to verify the transfection efficiency at different time points after 5-8F and SUNE-1 were transfected into miR-451a mimic or negative control (NC). ^**^*P*<0.001 vs. blank.
