# Supplementary figures and images for: The microRNA-451a/chromosome segregation 1-like axis suppresses cell proliferation, migration, and invasion and induces apoptosis in nasopharyngeal carcinoma
Source: Bioengineered. 2021 Sep 13;12(1):6967–80. doi: 10.1080/21655979.2021.1975018 (PMC8806603; doi:10.1080/21655979.2021.1975018)

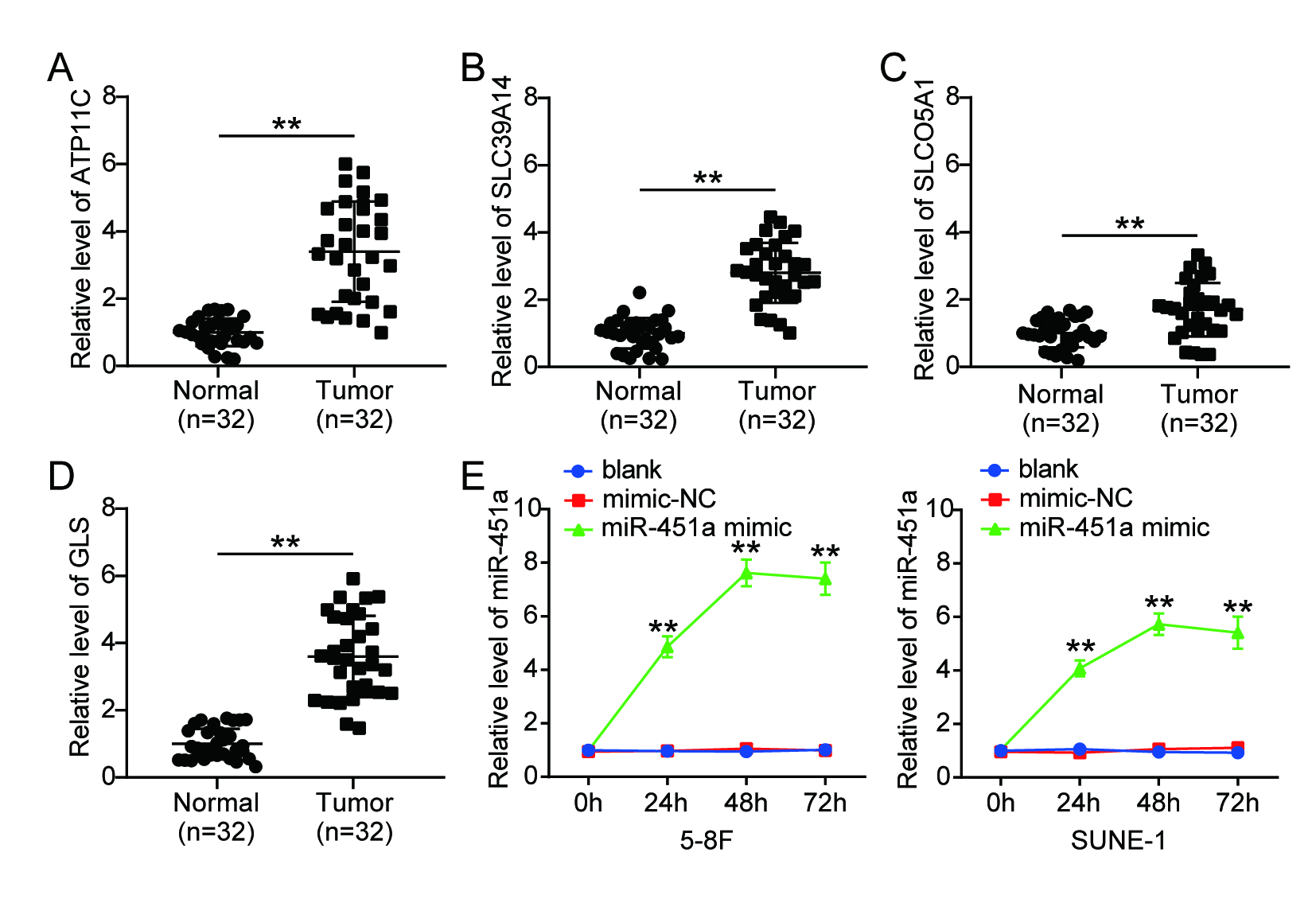

Supplement: Supplemental Material [file KBIE_A_1975018_SM2662.zip › supplementary/Supplementary figure 1_revised.tif]
